# Life-course socio-economic factors associated with frailty in later life^☆^

**DOI:** 10.1016/j.tjfa.2025.100107

**Published:** 2025-11-26

**Authors:** Mathilde Glud Christensen, Katja Kemp Jacobsen, Charlotte Juul Nilsson, Randi Jepsen, Lau Caspar Thygesen, Charlotte Suetta, Ellen Astrid Holm

**Affiliations:** a Zealand University Hospital, Køge, Medical Department, Geriatric section, Denmark; b Copenhagen Center for Clinical Research – CopenAge, Faculty of Health, University of Copenhagen, Denmark; c University College Copenhagen, Department of Technology, Faculty of Health and Technology, Denmark; d University of Copenhagen, Department of Public Health, Section of Social Medicine, Copenhagen, Denmark; e Lolland-Falster Health Study, Zealand University Hospital, Nykøbing F, Nykøbing F. Hospital, Denmark; f National Institute of Public Health, University of Southern Denmark, Denmark; g Geriatric Research Unit, Department of Geriatric and Palliative Medicine, Copenhagen University Hospital, Frederiksberg and Bispebjerg, Copenhagen, Denmark; h Copenhagen University Hospital – Steno Diabetes Center Copenhagen, Department for Clinical and Translational Research, Herlev, Denmark

**Keywords:** Frailty, Aging, Life-course, Socio-economic factors, Cumulative disadvantage

## Abstract

**Purpose:**

Frailty increases with age and is associated with negative health outcomes as falls, hospitalization, and mortality. Socio-economic situation (SES) in childhood and adulthood are associated with frailty. It is unclear how the interaction of childhood hardship and adulthood SES effects frailty.

**Methods:**

A register-based study using data from national registers and from the Lolland-Falster Health Study, involving individuals aged 50 and above. Frailty status was assessed using a modified version of Fried’s phenotype. Logistic regression models with multiple adjustments were used to analyze the odds of frailty. Causal interactions between economic hardship in adulthood, perception of childhood, self-reported stressful events in childhood, and self-reported educational level were assessed by estimating the relative excess risk due to interaction (RERI).

**Results:**

The study included 10,163 individuals. The percentage of individuals fulfilling 2–5 frailty criteria varied between 17 % in the 50–65 age group and 44.9 % in the 85+ age group. Women had a higher proportion of fulfilling 2–5 frailty criteria (21.5 %) compared to men (17.2 %). Socio-economic factors associated with frailty status included perception of childhood, stressful childhood events, educational attainment, and economic hardship in adulthood. A significant causal additive effect on the percentage of individuals fulfilling 2–5 frailty criteria was demonstrated for two composite outcomes: perception of childhood + educational attainment and stressful events in childhood + economic hardship in adulthood.

**Conclusion:**

The study showed that joint exposure to adverse socio-economic factors in childhood and adulthood, potentiated the odds of frailty in older adults. Our findings corroborate the theory of cumulative dis/advantage.

**Strengths and limitations of this study:**

This study combines self-reported data with high-quality national register data, enabling robust, longitudinal assessment of socioeconomic exposures across the life course.

Frailty was measured using a validated instrument based on the SHARE-FI scale, allowing for comparability with previous European studies.

Composite exposure variables were constructed to formally assess additive interaction and cumulative effects of early-life and adulthood socioeconomic factors.

Childhood exposures were self-reported retrospectively, which may introduce recall bias.

Economic hardship was measured over a 30-year period; however, variation in participant age means it may reflect different life stages (e.g. early adulthood vs retirement), complicating temporal interpretation.

Alt-text: Unlabelled box

## 1 Introduction

Frailty is defined as “a biologic syndrome of decreased reserve and resistance to stressors, resulting from cumulative decline across multiple physiologic systems, and causing vulnerability to adverse outcomes” [1] and may be used to explain the heterogeneity in ageing [1,2]. Although the research on frailty is substantial, a consensus on neither a screening tool nor a universal method of measuring frailty has been found. A commonly used method is Fried´s frailty phenotype (FP) [3,4]. The FP defines frailty as a clinical syndrome when meeting three or more of the following five phenotypic criteria: weakness, slowness, low level of physical activity, self-reported exhaustion, and unintentional weight loss. When meeting one or two criteria, the syndrome is characterized as prefrailty.

The prevalence of frailty is increasing, due to the ageing society, and is of importance for the individual and society in general because it is associated with negative health outcomes such as loneliness, falls and fractures, hospitalization, institutionalization, and increased mortality [[4], [5], [6], [7]]. The process of developing frailty begins early in life - possibly already in utero, and the influence of factors associated with frailty may accumulate throughout life [8].

Socio-economic status (SES) in adulthood such as living alone, low income, and low educational attainment are associated with frailty [[9], [10], [11], [12]]. Throughout the last decade, heightened attention to early modifiable risk factors for frailty has arisen. These include events or circumstances during childhood and adolescence, that independently or in combination increase the risk of frailty [13].

Childhood environment and stressful events in childhood have been shown to increase morbidity and mortality in adult life and thereby possibly accelerate ageing and increase the risk of frailty [[13], [14], [15]].

Although it has been shown that stressful events in childhood are associated with frailty in later life, it is unclear whether the effect of childhood events on frailty is mediated by circumstances in adulthood such as educational attainment, wealth, and behavioral factors or if they per se increase the risk of frailty [16,17]

Chronic diseases and behavioral factors such as smoking, body mass index and alcohol use have been suggested as possible inducers or aggravators of frailty [1,18,19]. Childhood adverse events have been shown to be associated with several acute and chronic diseases in adulthood including mental health, multimorbidity, chronic pain, obesity, diabetes, impaired cognition and dementia, hypertension, and cancer [[20], [21], [22], [23]]

Several possible mechanisms through which adverse childhood events may influence development and ageing have been suggested: chronic elevation of stress hormones; elevation of inflammatory markers; alterations in the hypothalamic-pituitary adrenal axis; neurodevelopmental changes; telomere shortening, indicating accelerated cell ageing; epigenetic changes; and allostatic overload [[24], [25], [26], [27]].

The aim of the present study is to assess how the combination of circumstances and events in childhood, in young adulthood, and SES factors in adulthood affect frailty in later life. Furthermore, we will study the possible interactions between the influence of childhood circumstances and adulthood SES on frailty.

The study draws on the theoretical framework “The Cumulative Disadvantage Theory”, which hypothesizes that disadvantages accumulate over time, with earlier life experiences and social factors compounding the effects of later life circumstances. By examining the interaction between childhood events, young adulthood experiences, and socio-economic status (SES) in adulthood, the study seeks to understand how these accumulated disadvantages contribute to frailty in later life.

## 2 Materials and methods

### 2.1 Data sources

Data were derived from the Lolland Falster Health Study’s (LOFUS) questionnaire and national registers. Every person residing in Denmark is registered with a unique personal identification number in the Danish Civil Registration System, which makes it possible to link several national registers. Personal identification numbers and demographic data were derived from the LOFUS database.

### 2.2 Lolland-Falster health study (LOFUS)

LOFUS is a household-based study where households of randomly selected persons aged 18 and above were invited to participate [28]. The data collection encompassed self-administered, age-specific questionnaires on social, mental and physical health and lifestyle factors, anthropometric and physiological measurements, collection of biological samples and a frailty measurement for participants ≥50 years. The data collection started in February 2016 and ended in February 2020.

### 2.3 Frailty

Frailty status was assessed using items almost identical to the Share-Frailty Instrument (SHARE-FI) developed from the SHARE study [29]. The SHARE-FI was chosen based on the similarity with the FP originally described by Fried and colleagues to ensure consistency with the existing literature [6]. In a previous study, we validated the frailty measurement used in LOFUS and found the Lolland-Falster Health Study frailty assessment being a valid instrument [30]. Details concerning the measurement of frailty status are shown in appendix Fig. 1.

**Fig. 1:**
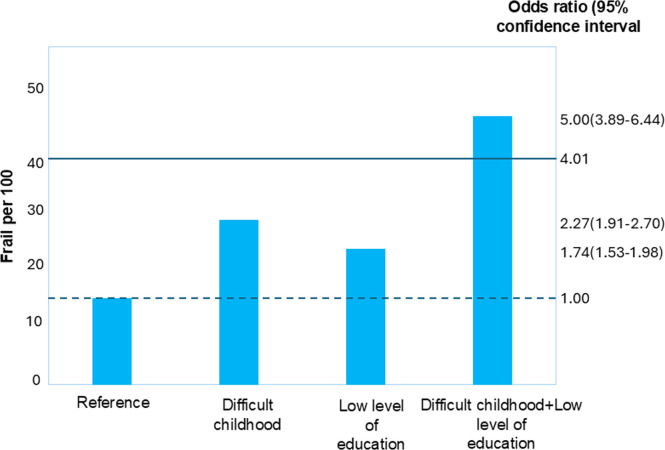
Composite outcomes of educational level and perception of childhood. The figure shows that low level of education combined with having had a difficult childhood leads to an excessive risk of frailty in later life. The dotted line indicates the background number of frail per 100 and odds = 1. The solid line indicates the exact additivity of the effects.

#### 2.3.1 Childhood and young adulthood socio-economic situation

Variables describing childhood and young adulthood were derived from the LOFUS questionnaire. The questionnaire contained four questions concerning stressful events in childhood answered with yes or no: Prolonged illness of parent(s); period of placement in foster care; prolonged family conflict; prolonged financial problems in the family. These items captured prolonged or severe family adversities rather than transient experiences.

“Prolonged parental unemployment” referred to continuous unemployment exceeding six months. Furthermore, one question assessed the overall perception of childhood with possible answers being very good, good, average, difficult, or very difficult. Data concerning educational attainment in young adulthood came from the LOFUS questionnaire and were classified into primary school only, and further education being short (1–3 years), medium (3–4 years), or long (> 4 years). Participants whose educational attainment did not fit these predefined national categories (e.g., foreign or unclassified vocational training) were categorized as “Other.

#### 2.3.2 Adulthood socio-economic situation

Economic hardship (EH) was defined as equivalized household disposable income (total yearly household income minus total taxes accounting for the number of people in the household) less than 60 % of the median household disposable income for the entire Danish population that year [31]. This corresponds to the European Union’s definition of people at risk of poverty [32]. EH was categorized as the number of years of economic hardship from 1988 to 2018 (0, 1–3, or ≥4 years). Yearly data on household income from 1988 to 2018 were attained from the national registry on income and transfer payments [33].

Household income was obtained from Statistics Denmark’s national administrative registers, which compile income data from mandatory tax reports for all residents. This ensures that income information is registry-based and not self-reported, thereby minimizing recall bias. This means that the participants were of various ages when the economic hardship was documented. Approximately one third of the participants (3270) were 50–59 years when included during 2016–2020, i.e. they were born between 1957 and 1970, and were between 18–31 years in 1988 when the recording of EH began. Only few of our participants (*n* = 579) were more than 80 years old, i.e. they were born before 1936 and were older than 52 when the recording of EH began. Thus, for the youngest of our participants, EH may reflect the situation in young adulthood and for the oldest, EH may reflect the economic situation during retirement.

#### 2.3.3 Potential confounders

From the LOFUS questionnaire, we included smoking (years with smoking categorized into 0; ≤10; 10–20; 20–30: >30) and historical weight (change between self-reported weight one year before participation in LOFUS and self-reported weight at age 20 years) categorized into weight stable ±5 kg, weight gain >5 kg or weight loss >5 kg. Comorbidity was assessed using data from the National Patient Register (covering the period 1994–2020) containing diagnoses registered according to the International Statistical Classification of Diseases and Related Health Problems, tenth revision (ICD-10) for all hospital contacts [34]. The included diagnoses were ischemic heart disease, hypertension, chronic kidney disease, cancer, depression, chronic obstructive pulmonary disease (COPD), and diabetes. These diagnoses were chosen due to their known association with frailty [6]. Details are shown in appendix, Table 1S.

Patient and Public involvement

Patients and the public were not involved in the design of this study.

### 2.4 Data analysis

We used descriptive statistics to show the distribution of socio-economic variables.

Logistic regression was used to assess the association of exposure variables and confounders with frailty status. Participants with 0–1 frailty criteria fulfilled were compared to those with 2–5 criteria fulfilled.

In model 1, the regression analysis was adjusted only for age-group and sex, model 2 was adjusted for all variables except comorbidity (historical weight, history of smoking, economic hardship, education, number of stressful events in childhood, and perception of childhood), and in model 3, we included adjustment for comorbidity.

Causal interaction is a theoretical concept defined as a deviation from the additivity of the absolute effects of the risk factors being studied [35]. To evaluate the presence of causal interaction between childhood experiences, economic hardship, and educational level on an additive scale, we created five new composite variables: 1) stressful events in childhood + economic hardship in adulthood; 2) stressful events in childhood + educational attainment in adolescence/young adulthood; 3) overall perception of childhood + economic hardship in adulthood; 4) overall perception of childhood + educational attainment in young adulthood; 5) stressful events in childhood + overall perception of childhood. The five composite variables were computed with four combinations, the joint reference category of no exposure (-/-), a category of exposure to one variable (-/+ or ±) and the joint exposure to both variables (+/+). For each composite variable and combination, we calculated the number of individuals with frailty per 100 persons. If interaction is present, the number of individuals with frailty within the group with joint exposure to both risk factors (+/+) should be higher than the sum of the remaining categories (-/-, -/+, ±).

Additionally, logistic regression was used to evaluate the odds of frailty using the composite variables. As recommended in the STROBE guidelines, [36] we report the separate effect of each exposure as well as the joint effect compared to the joint reference. The relative excess risk due to interaction (RERI) was calculated to quantify the amount of interaction [35,37]. RERI is calculated as the difference between the expected risk and the observed risk (RERI = OR_+_- OR_+-_ - OR_-+_ 1). In the absence of interaction, RERI equals 0.

Analyses were performed using STATA/SE 15.1 (StataCorp LLC, Texas, USA).

## 3 Results

LOFUS included 18,949 individuals, and 11,057 individuals were 50 years of age or above. Of these, 10,163 answered the questions on frailty. Nine individuals were excluded due to three or more missing frailty components, leaving 10,154 individuals for analysis.

The percentage of individuals fulfilling two-five frailty criteria varied between 17 % in the 50–65 age group and 44.9 % in the 85+ age group. Women had a higher proportion of fulfilling 2–5 frailty criteria (21.5 %) compared to men (17.2 %). The distribution of all variables is shown in Table 1.

**Table 1:** Distribution of socio-demographic variables. Distribution of socio-demographic variables among 10,154 participants with available data on frailty from LOFUS.

			Number		
Age	Years		Total (10,154)	0–1 frailty criteria (8180)	2–5 frailty criteria (1974)
		50–65 65–74 75–84 85+	5090 3623 1276 165	4224(83.0) 2966(82.0) 899(70.5) 91(55.2)	866(17.0) 657(18.1) 377(29.6) 74(44.9)
Sex		Women	5318	4174(78.5)	1144(21.5)
		Men	4836	4006(82.8)	830(17.2)
Historical weight		Weight stable ±≤5 kg	2274	1943(85.4)	331(14.5)
		Weight gain >5 kg	6179	4957(80.2)	1222(19,8)
		Weight loss >5 kg	389	292(75.1)	97(24,9)
		Missing	1312		
History of smoking	Years duration	Never smoker	4242	3613(85.2)	629(14.8)
		≤10	958	735(76.7)	192(22.9)
		10–20	1008	799(79.3)	202(20.8)
		20–30	730	616(84.0)	114(15.6)
		>30	2595	1957(75.4)	638(24.6)
		Missing	621		
Socio-economic status	Economic hardship, years	0	6872	5672(82.9)	1170(17.1)
		1–3	2123	1632(76.9)	491(23.1)
		4 or more	1159	854(73.7)	305(26.3)
	Educational level	Primary school	1017	669(65.8)	348(34.2)
		Short (1–3 years)	6030	4923(81.6)	1107(18.4)
		Medium (3–4 years)	2025	1718(84.8)	307(15.2)
		Long >4 years	413	361(87.4)	52(12.6)
		Other	617	479(77.6)	138(22.4)
		Missing	52		
Childhood	Stressful events in childhood and youth	Prolonged illness of parent(s) YES No Missing	2640 7455 59	2058(78.0) 6076 (81,5)	582(22.1) 1379 (18,5)
		Period of placement in foster care: YES NO Missing	574 9501 79	382(66.6) 7738(81.4)	192(33.5) 1763(18.6)
		Prolonged family conflict YES NO Missing	1455 8608 91	1080(74.2) 7035(81.7)	375(25.8) 1573 (18,3)
		Prolonged unemployment of parent(s): YES NO Missing	453 9605 96	315(69.5) 7797(81.2)	138(30.5) 1808(18.8)
		Prolonged financial problems in the family: YES NO Missing	1121 8907 126	796(71.0) 7295 (82)	325(29.0) 1612(18)
	Number of stressful events in childhood	0	6075	5079(83.6)	996(16.4)
		1	2634	2045(77.6)	589(22.4)
		2	927	707(76.3)	220(23.7)
		3 or more	518	349(67.4)	169(32.6)
	Perception of childhood	Good (very good + good)	7854	6504(82.8)	1350(17.2)
		Average	1434	1088(75.9)	346(24.1)
		Difficult (difficult +very difficult)	793	534(67.3)	259(32.7)
		Missing	73		

Exhaustion was the most frequent frailty component (41.7 %), followed by slowness (13.2 %) and low physical activity (12.2 %) (Table 2). Age, female sex, weight gain, economic hardship in adulthood, stressful childhood events, and perception of childhood as being average or difficult were associated with increased odds of fulfilling 2–5 frailty criteria.

**Table 2:** Prevalence of frailty criteria among 10,154 Danish individuals aged 50 years and above.

	Number (total with included frailty criteria)	Proportion ( %)	Proportion among men ( %)	Proportion among women ( %)
Frequency of frailty criteria				
Unintentional weight loss * (*n* = 10,073)	647	6.4	5.1	7.6
Exhaustion* (*n* = 10,073)	4211	41.7	39.1	44.1
Weakness *(*n* = 9710)	826	8.5	8.3	8.7
Slowness * (*n* = 10,144)	1334	13.2	11.8	14.4
Low activity * (*n* = 10,097)	1227	12.2	11.2	13.0

⁎ The number of frailty components vary due to missing values.

Smoking significantly increased the OR of fulfilling 2–5 frailty criteria (1.94, CI 1.71–2.20) in model 1 and 2 (1.84, CI 1.60–2.12) but was nonsignificant when comorbidity was included in the fully adjusted model (1.17, CI 0.95–1.44). In contrast, higher educational level lowered the odds of frailty (OR 0.33, CI 0.24–0.45 in model 1 and 0.46, CI 0.31–0.69 in the fully adjusted model). Hypertension, COPD, diabetes, and depression significantly increased the odds of frailty, with depression showing the strongest association (Table 3).

**Table 3:** Odds of fulfilling 2–5 frailty criteria versus fulfilling 0–1 criteria Odds of fulfilling 0–1 versus 2–5 frailty criteria among 10,154 individuals aged ≥50 years according to age, sex, socio-economic status, lifestyle status and comorbidity in the Lolland-Falster Health Study, Denmark.

	OR (95 %CI) Adjusted for age and sex	p-value	OR (95 %CI) Multi-adjusted model	p-value	OR (95 %CI) Multi-adjusted model +comorbidity	p-value
Age, years						
50–65 65–74 75–84 85+	1.00 1.09(0.98–1.22) 2.09(1.82–2.41) 4.04(2.94–5.54)	.13 <0.0001 <0.0001	1.00 1.13(0.97–1.32) 2.33(1.91–2.83) 5.53(3.58–8.54)	0.11 <0.0001	1.00 1.00(0.85–1.17) 1.93(1.57–2.37) 4.40(2.81–6.91)	0.99 < 0.0001 < 0.0001
Sex						
Men	1.00		1.00			
Women	1.36(1.23–1.50)	<0.0001	1.34(1.16–1.53)	<0.0001	1.40(1.21–1.61)	< 0.0001
Historical weight, kg						
Weight stable ±≤5	1.00		1.00		1.00	
Weight gain >5	1.57(1.37–1.80)	<0.0001	1.62(1.36–1.92)	<0.0001	1.56(1.31–1.86)	< 0.0001
Weight loss >5	1.84(1.41–2.39)	<0.0001	1.23(0.84–1.80)	.27	1.13(0.77–1.65)	0.55
History of smoking, years duration						
0	1.00				1.00	
≤10	1.95(1.64–2.32)	<0.0001	1.74(1.43–2.11)	<0.0001	1.60(1.30–1.97)	< 0.0001
10–20	1.60(1.34–1.91)	<0.0001	1.41(1.16–1.72)	<0.0001	1.29(1.05–1.59)	.014
20–30	1.12(0.90–1.39)	.32	1.04(0.81–1.32)	.77	1.02(0.79–1.31)	.89
>30	1.94(1.71–2.20)	<0.0001	1.84(1.60–2.12)	<0.0001	1.17(0.95–1.44)	.14
Economic hardship, years						
0	1.00		1.00		1.00	
1–3	1.40(1.24–1.58)	<0.0001	1.27(1.08–1.50)	.004	1.24(1.05–1.47)	.011
4 or more	1.71(1.48–1.98)	<0.0001	1.38(1.12–1.69)	.002	1.33(1.08–1.64)	.007
Education						
Primary school	1.00		1.00		1.00	
+ Short (1–3 years)	0.50(0.43–0.58)	<0.0001	0.60(0.49–0.75)	<0.0001	0.61(0.49–0.75)	< 0.0001
+ Medium (3–4 years)	0.39(0.33–0.47)	<0.0001	0.44(0.34–0.56)	<0.0001	0.43(0.34–0.56)	< 0.0001
+ Long >4 years	0.33(0.24–0.45)	<0.0001	0.45(0.30–0.66)	<0.0001	0.46(0.31–0.69)	< 0.0001
Other	0.60(0.48–0.76)	<0.0001^5^	0.72(0.52–1.00)	.05	0.71(0.51–1.00)	.045
Number of stressful events in childhood						
0	1.00		1.00		1.00	
1	1.50(1.33–1.68)	<0.0001	1.28(1.10–1.50)	.002	1.28(1.09–1.50)	.002
2	1.67(1.41–1.97)	<0.0001	1.30(1.03–1.64)	.027	1.27(1.00–1.61)	.05
3 or more	2.55(2.09–3.11)	<0.0001	1.47(1.09–1.98)	.012	1.42(1.05–1.93)	.02
Perception of childhood						
Good (very good + good)	1.00		1.00		1.00	
Average	1.58(1.37–1.81)	<0.0001	1.45(1.20–1.75)	<0.0001	1.41(1.16–1.70)	.0005
Difficult (difficult + very difficult)	2.43(2.07–2.86)	<0.0001	1.92(1.50–2.45)	.022	1.82(1.41–2.35)	< 0.0001
Comorbidity						
Hypertension					1.81(1.50–2.19)	< 0.0001
Ischemic heart disease					1.62(0.90–2.91)	.11
COPD					1.89(1.49–2.41)	< 0.0001
Diabetes					2.54(1.97–3.29)	< 0.0001
Depression					2.75(1.69–4.48)	< 0.0001
Chronic kidney disease					1.16(0.59–2.30)	.66
Cancer					1.21(0.98–1.48)	.06

Two composite outcomes showed interaction with frailty. Stressful events in childhood resulted in an excess number of frail individuals of 6.3 per 100 persons while economic hardship in adulthood resulted in an excess number of 5.5 per 100 persons. Overall perception of childhood as average/difficult resulted in an excess number of frail individuals of 13.9 and low educational attainment in an excess number of 8.7 frail individuals per 100 persons. For the composite outcome stressful events in childhood + economic hardship in adulthood, the OR for frailty was 0.44 higher than expected from the addition of separate effects, and for the overall perception of childhood as average/difficult + low educational attainment, the OR was 1.99 higher than expected (Table 4, Fig. 1 and Appendix, Table 2S). Results from the remaining composite outcomes are shown in Appendix, Table 3S- 5S.

**Table 4:** Composite outcomes of stressful events in childhood and economic hardship in adulthood.

	Total Number ( % with 2–5 frailty criteria fulfilled)	OR (95 %CI)	OR (95 %CI) Adjusted for age and sex	OR (95 %CI) Fully adjusted*
No Economic hardship + no stressful events in childhood (-/-)	4201(14.7)	1.00	1.00	1.00
No Economic hardship + stressful events in childhood (-/+)	2671(21.0)	1.53(1.35–1.75)	1.57(1.38–1.78)	1.18(1.01–1.37)
Economic hardship + no stressful events in childhood (±)	1874(20.2)	1.46(1.27–1.69)	1.41(1.21–1.63)	1.21(1.02–1.43)
Economic hardship + stressful events in childhood (+/+)	1408(29.7)	2.45(2.12–2.82)	2.46(2.13–2.85)	1.92(1.62–2.29)
Excess number (composite vs. single) per 100 cases	3.2			
RERI (95 %CI)		0.44(0.08–0.80)		

Table 3: Odds of having 2–5 frailty criteria versus 0–1 frailty criteria among 10 154 individuals aged ≥50 years.

The excess number of participants with 2–5 frailty criteria fulfilled per 100 persons was calculated as the difference between the actual number and the expected number. In individuals without stressful events in childhood and without economic hardship during adulthood the number of individuals with frailty was 14.7 per 100, which is considered the background number. In individuals with stressful events, the number increased by 6.3 (21.0–14.7), and in individuals with economic hardship, the number increased by 5.5 (20.2–14.7). In individuals with both stressful events in childhood and later economic hardship the number was 29.7, which means that the observed number is 3,2 higher per 100 persons than the expected number of 26.5 (14.7 + 5.5 + 6.3). Based on the crude estimates from the logistic regression, the relative excess risk due to interaction was 0.44, 95 % CI (0.08–0.80) indicating presence of interaction between stressful events in childhood and economic hardship in adulthood.

⁎ Adjusted for the remaining variables in the final model.

## 4 Discussion

In this study, we investigated associations between frailty and socio-economic situation in childhood (overall perception of childhood and stressful events in childhood), in adolescence and young adulthood (educational attainment), and in adulthood (number of years with economic hardship in the period 1988–2018). An important finding was associations between the number of frailty criteria fulfilled and age, female sex, weight gain, economic hardship, stressful childhood events, and overall perception of childhood. In contrast, higher education was found to protect against frailty.

Additionally, we assessed the effect of joint exposure as relative excess risk due to interaction (RERI) among factors along the life course. A significant additive effect on the risk of frailty was demonstrated for the overall perception of average/difficult childhood in combination with low level of educational attainment (adolescence/young adulthood) and for stressful events in childhood in combination with economic hardship in adulthood.

### 4.1 Frailty status

Fried et al. classified frailty status into non-frail/robust with 0 frailty criteria, pre-frail with 1–2 frailty criteria and frail with 3–5 criteria (6). Instead of using this classification, we compared participants with 0–1 frailty criteria to participants with 2–5 criteria fulfilled. In our study, almost half the participants (41.7 %) fulfilled the frailty criteria of exhaustion while only 17 % fulfilled this criteria in the original study by Fried et al. [6] Based on this difference we assumed that fulfillment of one criterion in our population and with our measurement instrument might not be indicative of prefrailty. On the other hand, it has been shown that prefrailty is associated with the same adverse events as frailty [6,38]. Based on these reflections we chose to compare participants with 0–1 frailty criteria to participants with 2–5 criteria fulfilled.

### 4.2 Weight change and frailty

Although unintentional weight loss is often considered a component of frailty, our study's findings suggest a more complex relationship between weight changes and frailty. Specifically, the observation that weight gain of more than 5 kg was associated with an elevated risk of frailty (OR 1.56) highlights the potential role of sarcopenic obesity, where increased fat mass exacerbates inflammation and decreases muscle function, potentially leading to reduced mobility and increased frailty.

Furthermore, the lack of association between weight loss exceeding 5 kg and frailty might be influenced by various biases. Self-reported weight changes are susceptible to inaccuracies, particularly among older populations with cognitive challenges. Severe or illness-induced weight loss might have led to non-response due to mortality or severe frailty, thus underrepresenting the frailest individuals. Additionally, including cases of intentional weight loss in our data may have further diluted the anticipated correlation between weight loss and frailty. These factors necessitate cautious interpretation and indicate the need for more precise measurement tools in future research to clarify the nuances of weight change impacts on frailty risk.

### 4.3 Smoking and frailty

The unexpected finding that light smoking, defined as less than 10 cigarettes per day, was associated with a higher risk of frailty compared to heavier smoking, can likely be attributed to survivor bias, reverse causation, or mortality bias. Individuals who were more frail and experiencing health decline may have reduced smoking or quit, thus reflecting a different smoking behavior among frailer individuals. Additionally, heavier smokers might have faced higher mortality rates due to smoking-related diseases, resulting in an underrepresentation of this group in the study. Consequently, the observed association may not accurately reflect the direct effects of smoking levels on frailty but rather an interplay of survivor effects and underlying health conditions.

### 4.4 Socio-economic situation in adulthood

Our finding that economic hardship and educational attainment were associated with frailty is in accordance with findings in many other published studies [18,39,40]. For most of the participants in our study, the period with economic hardship was recorded in the national registers many years before the diagnosis of frailty supporting the hypothesis that socio-economic inequalities have long-lasting effects on health in older age.

### 4.5 Childhood circumstances

Stressful events in childhood, overall perception of childhood, and low educational attainment were significantly associated with frailty prevalence.

This is in accordance with previous findings [8,16,41]. In our study, these effects did not disappear when adjusted for socio-economic situation in adulthood and were still significant in the fully adjusted model. This indicates that the effect of childhood circumstances on the development of frailty is not solely mediated by socio-economic factors in adulthood. There are inconsistencies when comparing our study to other studies and between studies. Some studies found that the effect of socio-economic variables in childhood disappeared when adulthood SES variables were included [17] and other studies found that the effect of socio-economic parameters in adulthood explained a large part of the effect of childhood SES-parameters on frailty [18]. These differences can be explained by various ways of defining childhood SES-variables e.g. household income, occupation of breadwinner, housing, number of books in the home etc. and by different ways of measuring stressful events in childhood. Adverse childhood events have been defined as “potentially traumatic events, including maltreatment, abuse, and harmful environments” and have been measured with several instruments [42]. In our study, the items concerning the socio-economic situation in childhood were included as two of five questions describing stressful events in childhood (prolonged unemployment of a parent and prolonged financial troubles). Previous studies exploring the associations between childhood circumstances and frailty are mainly cross-sectional population studies and birth cohort studies and the specific items included have varied [8,16,17,[43], [44], [45], [46]]. However, common for these studies is that childhood circumstances were associated with frailty, but the degree to which the effect was explained by adulthood SES-factors varied.

### 4.6 Joint exposures

We found significant causal interactions between the single effects included in two composite variables: stressful events in childhood + economic hardship in adulthood and overall perception of childhood+ educational attainment. In other words, having had both exposures in one of these combinations resulted in a larger-than-expected prevalence of frailty. These findings support the theory of cumulative dis/advantage, which explains the diversity in ageing partly as a result of differences in dis/advantages cumulated over a life course [47].

Our findings demonstrate an additive effect of early-life adversity and later economic hardship on frailty risk, emphasizing that prevention should begin early in life. Clinically, incorporating socioeconomic history into geriatric assessment could help identify individuals at increased risk of frailty.

### 4.7 Confounders

Comorbidity and lifestyle modified and partly explained the association between frailty and childhood circumstances as well as SES in adulthood (model 2 and 3 in Table 3). This has been demonstrated in several previous studies [17,18].

Future studies in other welfare and cultural contexts are needed to test whether the cumulative disadvantage pathway operates similarly across different social and economic systems.

### 4.8 Strengths and limitations

The study used validated methods for measuring frailty, including SHARE-FI, a modified version of Fried’s phenotype [30]. The study involved a large population sample, providing detailed information on individual experiences including childhood adversities and economic challenges. Approximately 41 % of those invited who were 50 years or above chose to participate, which aligns with similar research [23,39]. However, participation was lower among the oldest individuals and those with lower socio-economic status, which may result in an underestimation of frailty prevalence among the oldest participants [48].

The effect of childhood disadvantage on frailty may also be underestimated due to selection bias. Childhood disadvantage has been shown to be associated with increased morbidity and mortality in adulthood, and exposed individuals may therefore not live long enough to become frail.

We cannot exclude that the information concerning stressful events in childhood and overall perception of childhood suffer from recall bias. However, previous studies comparing register-based information with information obtained from elderly people have shown recall data to be a reliable measure [49,50].

## 5 Conclusion

This study highlights the multifactorial nature of frailty, showing that socio-economic and psychosocial factors, and diseases over a life course influence prevalence of frailty in older adults.

Age, female sex, weight gain, economic hardship, and stressful childhood were all associated with increased odds of frailty, whereas higher education was protective against frailty. A significant causal additive effect on the risk of frailty was demonstrated for the combination of perception of difficult childhood + low educational level and the combination of stressful events in childhood + economic hardship in adulthood. Our findings corroborate the theory of cumulative dis/advantage.

## Ethical approval

Ethics approval statement: Informed written consent was obtained from all LOFUS participants. The LOFUS study was approved by the Region Zealand’s Ethical Committee on Health Research (SJ-421) and the Danish Data Protection Agency (p-2024–16360). The present study is registered under the Danish Data Protection Agency (P-2019–191). LOFUS is registered in Clinical Trials (NCT02482896).

## Author contributions

Randi Jepsen provided the LOFUS data, Lau Caspar Thygesen, Charlotte Suetta and Charlotte Juul Nilsson contributed to interpretation of data analysis. Katja Kemp Jacobsen made substantial contributions in designing and performing the statistical analysis. Ellen Astrid Holm contributed to the design of the study and interpretation of data, and Mathilde Glud Christensen contributed to the design of the study, the interpretation of data analysis, and made the first draft of the article. All authors performed critical revision of the article draft, approved the final version, and agreed to be accountable for all aspects of the work.

## Funding statement

The study received funding from Region Zealand. The funding source had no influence on study design, data analysis, or interpretation.

## Data sharing statement

Data are available upon reasonable request from Lolland-Falster Health Study, Statistics Denmark and the Danish Health Data Authority following Danish legislation.

## Declaration of generative AI and AI-assisted technologies in the writing process

Generative AI tools were solely used to correct and enhance the quality of language and formulation in the manuscript. No AI was involved in the scientific content, data analysis, or interpretation.

## CRediT authorship contribution statement

**Mathilde Glud Christensen:** Writing – review & editing, Writing – original draft, Methodology, Conceptualization. **Katja Kemp Jacobsen:** Writing – review & editing, Formal analysis. **Charlotte Juul Nilsson:** Writing – review & editing, Supervision. **Randi Jepsen:** Writing – review & editing. **Lau Caspar Thygesen:** Writing – review & editing, Supervision. **Charlotte Suetta:** Writing – review & editing, Supervision. **Ellen Astrid Holm:** Writing – review & editing, Methodology, Funding acquisition, Conceptualization.

## Declaration of competing interest

The authors declare that they have no known competing financial interests or personal relationships that could have appeared to influence the work reported in this paper.

## Appendix Supplementary materials

Image, application 1
