# Supplementary material for: Life-course socio-economic factors associated with frailty in later life
Source: J Frailty Aging. 2025 Nov 26;14(6):100107. doi: 10.1016/j.tjfa.2025.100107 (PMC12790040; doi:10.1016/j.tjfa.2025.100107)
Supplement: Supplementary file 1 [file mmc1.docx]

**Appendix**

Table of content

[Figure 1, Frailty measurement 2](#_Toc190417691)

[Table 1S: Diagnoses used to adjust for chronic diseases 3](#_Toc190417692)

[Table 2S: Composite outcomes of education and overall perception of childhood. 4](#_Toc190417693)

[Table 3S: Composite outcomes of education and stressful events in childhood. 5](#_Toc190417694)

[Table 4S: Composite outcomes of perception of childhood and economic hardship in adulthood. 6](#_Toc190417695)

[Table 5S: Composite outcomes of overall perception of childhood and stressful events in childhood. 7](#_Toc190417696)

## Figure 1, Frailty measurement

| The frailty measurement includes the following variables:  1) Exhaustion/Fatigue: the criterion was fulfilled by answering yes in response to the question “In the last month or so, have you had too little energy to do things you wanted to do?” (Yes/No).  2) Shrinking: the criterion was fulfilled by answering yes in response to the question “What has your appetite been like? Do you feel a diminution in desire for food?” (Yes/No).  3) Weakness was derived from the highest of three consecutive dynamometer measurements of handgrip strength in the dominant hand applying gender and body mass index cutoffs set by Fried and associates. [1]  4) Slowness: A positive answer to either of the following two items “Because of a health problem, do you have difficulty [expected to last more than 3 months] walking 100 meters” or “climbing one flight of stairs without resting”.  5) Low physical activity was fulfilled in participants responding one to three times a month, hardly ever, or never to the question “How often do you engage in activities that require a low or moderate level of energy such as gardening, cleaning the car, or going for a walk?”  1) Fried, L.P., et al., *Frailty in older adults: evidence for a phenotype.* J Gerontol A Biol Sci Med Sci, 2001. **56**(3): p. M146-56. |
| --- |

## Table 1S: Diagnoses used to adjust for chronic diseases

|  | ICD-10 codes |
| --- | --- |
| Ischemic heart disease | DI20-DI25 |
| Hypertension | DI10-DI15 |
| Chronic kidney disease | DN17-DN19 |
| Cancer | DC00-DC96 |
| Depression | DF32-DF34 |
| COPD | DJ40-DJ47 |
| Diabetes | DE10-DE14 |

## Table 2S: Composite outcomes of education and overall perception of childhood.

|  | Total Number  (% with 2-5 frailty criteria fulfilled) | OR  (95%CI) | OR  (95%CI)  Adjusted for age and sex | OR  (95%CI)  Fully adjusted* |
| --- | --- | --- | --- | --- |
| Education beyond primary school + perception of childhood as good/very good  (-/-) | 6,613(15.4) | 1.00 | 1.00 | 1.00 |
| Education beyond primary school + perception of childhood as average/difficult (-/+) | 444(29.3) | 2.27(1.91-2.70) | 1.93(1.62-2.30) | 1.83(1.48-2.26) |
| No education beyond primary school + perception of childhood as good/very good  (+/-) | 1,809(24.1) | 1.74(1.53-1.98) | 1.78(1.57-2.03) | 1.54(1.32-1.80) |
| No education beyond primary school + perception of childhood as average/difficult (+/+) | 260(47.7) | 5.00(3.89-6.44) | 4.60(3.56-5.95) | 4.00(2.90-5.50) |
| Excess number (composite vs. single) per 100 cases | 9.7 |  |  |  |
| RERI |  | 1.99(0.70-3.27) |  |  |

*Adjusted for the remaining variables in the final model

Table 4: Odds of fulfilling 0-1 versus 2-5 frailty criteria among 10 154 individuals in the Lolland-Falster Health Study, Denmark aged ≥50 years. The excess number of participants with 2-5 frailty criteria fulfilled per 100 persons was calculated as the difference between the actual number and the expected number. The excess number due to perception of childhood as average/difficult was 29.3-15.4=13.9; the excess number due to no education beyond primary school was 24.1-15.4=8.7; the actual number of frail individuals with economic hardship + stressful childhood events was 47,7, which is 9.7 higher than the expected number of 38 (15.4+13.9+8.7).The relative excess risk due to interaction (RERI) was calculated to quantify the amount of interaction. RERI is calculated as the difference between the expected risk and the observed risk (RERI = OR_++_ - OR_+-_ - OR_-+_ +1). In the absence of interaction, RERI equals 0. Based on the crude estimates from the logistic regression, the relative excess risk due to interaction was 1.99 indicating presence of interaction between educational attainment and perception of childhood.

## Table 3S: Composite outcomes of education and stressful events in childhood.

| Var 3 | Total Number  (% with 2-5 frailty criteria fulfilled) | OR | OR  Adjusted for age and sex | OR  Fully adjusted* |
| --- | --- | --- | --- | --- |
| No stressful events in childhood + education beyond primary school (-/-) | 5,089(14.3) | 1.00 | 1.00 | 1.00 |
| No stressful events in childhood + No education beyond primary school (-/+) | 599(29.7) | 2.53(2.09-3.06) | 2.10(1.73-2.56) | 1.92(1.51-2.44) |
| Stressful events in childhood +education beyond primary school (+/-) | 3,379(21.8) | 1.67(1.49-1.87) | 1.69(1.50-1.89) | 1.33(1.16-1.52) |
| Stressful events in childhood + No education beyond primary school (+/+) | 418(40.7) | 4.10(3.32-5.06) | 3.76(3.03-4.66) | 2.83(2.17-3.70) |
| Excess number (composite vs. single) per 100 cases | 3.5 |  |  |  |
| RERI (95% CI) |  | 0.90(-0.02-1.83) |  |  |

*Adjusted for the remaining variables in the final model

Table S1: Composite outcomes of education and stressful events in childhood among 10,154 individuals aged >50 years. The excess number of participants with 2-5 frailty criteria fulfilled per 100 persons was calculated as the difference between the actual number and the expected number. The excess number due to no education beyond primary school was 29.7-14.3=15.4 ;The excess number due to stressful events was 21.8-14.3=7.5; the actual number of frail individuals with no education beyond primary school + stressful childhood events was 40,7, which is 3.5 higher than the expected number of 37.2 (14.3+15.4+7.5) .The relative excess risk due to interaction (RERI) was calculated to quantify the amount of interaction. RERI is calculated as the difference between the expected risk and the observed risk (RERI = OR_++_ - OR_+-_ - OR_-+_ +1). In the absence of interaction, RERI equal 0.

## Table 4S: Composite outcomes of perception of childhood and economic hardship in adulthood.

| Var 4 | Total Number  (% with 2-5 frailty criteria fulfilled) | OR | OR  Adjusted for age and sex | OR  Fully adjusted* |
| --- | --- | --- | --- | --- |
| No Economic hardship+ perception of childhood as good/very good (-/-) | 5,425(15.2) | 1.00 | 1.00 | 1.00 |
| No Economic hardship+ perception of childhood as average/difficult(-/+) | 1,403(24.2) | 1.77(1.53-2.05) | 1.83(1.58-2.11) | 1.64(1.38-1.95) |
| Economic hardship+ perception of childhood as good/very good (+/-) | 2,429(21.6) | 1.53(1.36-1.73) | 1.48(1.31-1.68) | 1.41(1.23-1.63) |
| Economic hardship + perception of childhood average/difficult (+/+) | 824(32.3) | 2.65(2.25-3.13) | 2.69(2.28-3.18) | 2.22(1.81-2.72) |
| Excess number (composite vs. single) per 100 cases | 1.7 |  |  |  |
| RERI (95% CI) |  | 0.35(-0.12-0.82) |  |  |

*Adjusted for the remaining variables in the final model

Composite outcomes of economic hardship and overall perception of childhood among 10,154 individuals aged >50 years. The excess number of participants with 2-5 frailty criteria fulfilled per 100 persons was calculated as the difference between the actual number and the expected number. The excess number due to difficult childhood was 24.2-15.2=9.0 ;The excess number due to economic hardship was 21.6-15.2=6.4; the actual number of frail individuals with difficult childhood + economic hardship in adulthood was 32.3, which is 1.7 higher than the expected number of 30.6 (15.2+9+6.4=30.6) .The relative excess risk due to interaction (RERI) was calculated to quantify the amount of interaction. RERI is calculated as the difference between the expected risk and the observed risk (RERI = OR_++_ - OR_+-_ - OR_-+_ +1). In the absence of interaction, RERI equals 0.

## Table 5S: Composite outcomes of overall perception of childhood and stressful events in childhood.

| Var 5 | Total number (% with 2-5 frailty criteria fulfilled) | OR | OR  Adjusted for age and sex | OR  Fully adjusted* |
| --- | --- | --- | --- | --- |
| No stressful events in childhood + perception of childhood as good/very good (-/-) | 5,321(15.4) | 1.00 | 1.00 | 1.00 |
| No stressful events in childhood + perception of childhood as average/difficult (-/+) | 2,533(21.0) | 1.47(1.30-1.66) | 1.50(1.33-1.70) | 1.40(1.21-1.61) |
| Stressful events in childhood + perception of childhood as good/very good (+/-) | 713(23.8) | 1.73(1.43-2.08) | 1.78(1.47-2.16) | 1.86(1.49-2.32) |
| Stressful events in childhood + perception of childhood average/difficult (+/+) | 1,514(28.7) | 2.22(1.94-2.54) | 2.33(2.03-2.67) | 2.18(1.87-2.55) |
| Excess number (composite vs. single) per 100 cases | -0.7 |  |  |  |
| RERI(95%CI) |  | 0.03(-0.39-0.45) |  |  |

*Adjusted for the remaining variables in the final model.

Composite outcomes of perception of childhood and stressful events in childhood among 10,154 individuals aged >50 years. The excess number of participants with 2-5 frailty criteria fulfilled per 100 persons was calculated as the difference between the actual number and the expected number. The excess number due to difficult childhood was 21.0-15.4=5.6 ; The excess number due to stressful events in childhood was 23.8-15.4=8.4; the actual number of frail individuals with difficult childhood + stressful events in childhood was 28.7, which is 0.7 less than the expected number of 29.4 (15.4+5.6+8.4=29.4) .The relative excess risk due to interaction (RERI) was calculated to quantify the amount of interaction. RERI is calculated as the difference between the expected risk and the observed risk (RERI = OR_++_ - OR_+-_ - OR_-+_ +1). In the absence of interaction, RERI equals 0.
